# Dysregulated Fatty Acid Metabolism in Preeclampsia Among Highland Andeans: Insights Into Adaptive and Maladaptive Placental Metabolic Phenotypes

**DOI:** 10.1096/fj.202502590R

**Published:** 2025-11-22

**Authors:** Katie A. O'Brien, Lilian Toledo‐Jaldin, Wanjun Gu, Julie A. Houck, Litzi Lazo‐Vega, Valquiria Miranda‐Garrido, Hong W. Yung, Hussna Yasini, Lorna G. Moore, Julie A. Reisz, Tatum S. Simonson, Jonathan Shortt, Margaret Stalker, Angelo D'Alessandro, Colleen G. Julian

**Affiliations:** ^1^ Department of Biomedical Informatics University of Colorado School of Medicine Aurora Colorado USA; ^2^ Centre for Human and Applied Physiological Sciences King's College London London UK; ^3^ Department of Obstetrics Hospital Materno‐Infantil La Paz Bolivia; ^4^ Division of Pulmonary, Critical Care, Sleep Medicine and Physiology, Department of Medicine University of California San Diego San Diego California USA; ^5^ Department of Neurology, Weill Institute for Neurosciences University of California, San Francisco San Francisco California USA; ^6^ Division of Reproductive Sciences, Department of Obstetrics and Gynecology University of Colorado School of Medicine Aurora Colorado USA; ^7^ Department of Physiology, Development and Neuroscience University of Cambridge Cambridge UK; ^8^ Department of Obstetrics and Gynaecology University of Cambridge Cambridge UK; ^9^ Department of Biochemistry and Molecular Genetics University of Colorado School of Medicine Aurora Colorado USA; ^10^ University of Colorado School of Medicine Anschutz Medical Campus Aurora Colorado USA

**Keywords:** adaptation, hypertensive disorders of pregnancy, hypoxia, metabolome

## Abstract

High‐altitude pregnancy presents the complex physiological challenge of fulfilling maternal, placental, and fetal metabolic demands under chronic ambient hypoxia. Highland Andeans exhibit signs of adaptation to high‐altitude hypoxia, showing relative protection against altitude‐associated fetal growth restriction (FGR) and the positive selection of metabolic genes linked to placental mitochondrial capacity. Not all infants are protected, with both FGR and preeclampsia occurring among highland‐resident Andeans. In Andeans, placental metabolic dysfunction is evident. By integrating metabolomic studies of maternal‐placental‐fetal triads with adaptive genetic signals in the fetal genome, we sought to identify adaptive and maladaptive placental metabolic phenotypes in highland Andeans (La Paz, Bolivia; 3850 m), including normotensive and preeclamptic pregnancies. Widespread differences in metabolite abundance were evident between normotensive and preeclamptic pregnancy across maternal, placental, and fetal compartments. Preeclampsia was characterized by a pronounced accumulation of fatty acid derivatives, specifically medium and long‐chain acylcarnitines; these were also associated with low birth weight. Genotype–phenotype association analyses revealed novel links between putatively adaptive fetal haplotypes and placental metabolite abundance. Carriers of specific adaptive fetal haplotypes comprising genes linked to lipid metabolism had a greater abundance of placental short‐chain acetyl‐carnitine alongside decreased levels of linolenic acid (*CPT2/LRP8*), lower levels of the medium‐chain octanoylcarnitine (*EXOC4*), and greater abundance of free carnitine (*LIPG*). Collectively, our study reveals a distinct metabolic phenotype in Andean preeclampsia characterized by incomplete fatty acid oxidation and highlights novel links between putatively adaptive fetal haplotypes and healthy placental metabolic phenotypes.

## Graphical abstract

Multi‐omic profiling of highland Andeans reveals adaptive fetal haplotypes associated with favorable placental metabolic phenotypes, while preeclampsia is marked by dysregulated fatty acid oxidation and acylcarnitine accumulation across maternal‐placental‐fetal compartments. Metabolomic profiling showed widespread accumulation of medium‐ and long‐chain acylcarnitines in preeclamptic cases versus normotensive controls, representing maladaptive and adaptive phenotypes, respectively. Adaptive fetal haplotypes, consisting of genes associated with lipid metabolism (*CPT2/LRP8*, *EXOC4*, *LIPG*) identified via integrative haplotype scores (iHS), were associated with healthier placental metabolic profiles. *Created in BioRender* (https://BioRender.com/j98qv87).

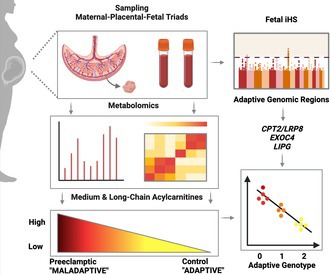

## 1 Introduction

Human populations have evolved to survive and reproduce across a remarkably diverse range of environments. High altitude is among the most physiologically demanding of these environments, where reduced atmospheric pressure limits oxygen availability (hypoxia) and imposes metabolic stress. Adaptation to environmental hypoxia necessitates physiological and molecular changes to preserve mitochondrial ATP production and redox balance [1, 2]. In the Andes, populations have lived at altitudes exceeding 3000 m for millennia [3], during which time natural selection has acted on genetic loci linked to oxygen sensing, cardiovascular function, and—critically—metabolic regulation [2, 4, 5, 6, 7, 8].

Since successful reproduction drives evolutionary fitness and is essential for population survival and growth, traits promoting positive pregnancy outcomes under chronic hypoxia are expected to be strongly selected in highland populations. High altitude challenges reproductive success partly due to the difficulty of meeting the metabolic demands of pregnancy in conditions of chronic ambient hypoxia, resulting in a gradual reduction in birth weight [9]. Birth weight is a crucial determinant of neonatal or infant mortality and a predictor of adverse health in later life [10, 11], making it a vital indicator of reproductive success. Andean ancestry confers partial protection from altitude‐associated reductions in birth weight resulting from fetal growth restriction (FGR), an adaptation attributed to greater fetal oxygen delivery [12] and more efficient placental and fetal oxygen utilization [13, 14]. Our previous work identified evidence of selective pressure on metabolic genes in the maternal genome that influenced placental mitochondrial respiratory capacity and, in turn, increased oxygen delivery to the fetus [15]; these findings support placental metabolic adaptations in Andean highlanders that may be genetic in nature.

Despite these population‐level adaptations, not all Andeans are protected from the negative impacts of high‐altitude gestation, and the incidence of pregnancy disorders such as preeclampsia—a placental disorder characterized by maternal hypertension, endothelial dysfunction, and fetal compromise—is elevated at high altitude [16, 17]. Given the evolutionary cost of maternal and perinatal mortality associated with preeclampsia [18, 19], physiological attributes that protect against this disorder under hypoxic conditions would be subject to intense selective pressure. Therefore, highland populations present a unique natural experiment for investigating the evolutionary dynamics of placental adaptation and the adverse consequences that occur when such adaptations fail.

Preeclampsia is marked by placental hypoxia and widespread metabolic dysfunction, particularly in lipid metabolism. Affected pregnancies show altered maternal and fetal (umbilical cord) plasma lipid profiles, including increased atherogenic indices such as higher lipid to lipoprotein ratios [20, 21, 22], and increased acylcarnitines—markers of impaired mitochondrial fatty acid transport—in maternal plasma [23, 24]; together, these signal dysregulated fatty acid oxidation. Decreased fatty acid oxidation enzyme expression [20, 25] and palmitate oxidation [25] are also evident in the preeclamptic placenta. As fatty acid oxidation critically depends on mitochondrial respiratory capacity, such findings point to a core role for mitochondrial dysfunction in the pathophysiology of preeclampsia. Our prior work in Andean placentas demonstrated impaired mitochondrial respiration at term in preeclamptic pregnancies and a decoupling of respiratory capacity from fetal oxygen delivery [15]. Moreover, preeclampsia activates the placental mitochondrial stress response, or unfolded protein response [26], which has been linked to suppressed mitochondrial respiratory capacity in trophoblast‐like BeWo cells [26]. Further supporting a central role for placental mitochondrial stress in preeclampsia, altered expression of mitochondrial respiratory complexes and markers of fission and fusion are evident in preeclamptic patients [27].

Understanding how these metabolic disruptions intersect with evolutionary adaptations to high altitude may illuminate both protective and pathological trajectories of placental function and provide deeper insight into the mechanisms underlying Andean adaptation to high altitude. Because preeclampsia is considered a placental disorder [28] and the placenta is fetal in origin, we propose that fetal genetics are directly involved in shaping these placental metabolic phenotypes. Recent findings that link fetal genetic variants to the risk of preeclampsia in a Peruvian highland population support this proposition [29].

In summary, evidence suggests that alterations to metabolic function during gestation are critical to Andean adaptation at high altitudes and that widespread metabolic disruption may underlie the etiology of preeclampsia. Here, we use metabolomic profiles of highland Andean maternal‐placental‐fetal triads to identify metabolic features distinguishing preeclamptic cases from normotensive controls. We further link these metabolic phenotypes to fetal haplotypes showing evidence of positive selection, providing insight into the evolutionary landscape of adaptive placental metabolic signals.

## 2 Methods

### 2.1 Study Population and Design

Participants were a subset of highland Andean mother‐infant pairs from a study investigating links between preeclampsia and neonatal pulmonary hypertension at high altitude [16]. All enrolled participants were delivered by scheduled, unlabored caesarean section at Hospital Materno‐Infantil in La Paz, Bolivia (3850 m). Admixture analysis using single‐nucleotide polymorphism (SNP) array data and *ADMIXTURE* [30] assuming four (*K* = 4) ancestral populations, indicated the cohort was predominantly of highland Amerindian origin (88%) with minimal European (6%), African (2%), and lowland Amerindian (5%) contributions [15]. The present study included 63 mother‐infant pairs (30 preeclamptic, 33 controls) selected from the original 79 based on the availability of placental and/or plasma samples for metabolomic profiling.

All participants provided written informed consent. Study procedures complied with the Declaration of Helsinki and were approved by the University of Colorado Multiple Institutional Review Board (Approval No. 18‐0210) and its Bolivian equivalents operated by the *Caja Nacional de Salud* and its Hospital Materno‐Infantil.

Preeclampsia diagnoses adhered to American College of Obstetrics and Gynecology [31] criteria: new‐onset hypertension after 20 weeks' gestation (≥ 2 systolic blood pressure [BP] measurements ≥ 140 mmHg or diastolic BP ≥ 90 mmHg at least 4 h apart or > 1 systolic BP ≥ 160 mmHg or diastolic BPs ≥ 110 mmHg) with at least one of the following symptoms: proteinuria, thrombocytopenia, impaired liver function, renal insufficiency, cerebral or visual disturbances, or pulmonary edema. Controls were required to be normotensive throughout pregnancy and postpartum. Inclusion criteria for all participants included maternal age between 18 and 45 years at enrollment, singleton pregnancy, permanent residence at high altitude for the duration of the current pregnancy, and no significant comorbidities (e.g., diabetes [type I or II], chronic hypertension, collagen vascular disease, cardiopulmonary disease), no evidence of moderate‐to‐severe anemia [hemoglobin < 8.5 g/dL].

At enrollment, information regarding maternal age, race/ethnicity, reproductive and health history (including prior placental complications, such as placenta previa, placenta accreta, or retained placenta), residential history, education, and marital status was obtained from medical records or by questionnaire. Prenatal records provided data on maternal BP, laboratory results, and pregnancy complications. Delivery and newborn data—including complications, birth weight, gestational age, infant sex, length, head circumference, and Apgar scores—were extracted from hospital records. Preterm birth was defined as delivery before 37 weeks' gestation. Small‐for‐gestational age (SGA) was defined as birth weight below the 10th percentile for gestational age and sex [32].

### 2.2 Biological Samples

Fasted maternal peripheral blood (8 mL) was collected via routine antecubital venipuncture into EDTA‐coated collection tubes within 4 h before delivery. Plasma was separated and snap‐frozen in liquid nitrogen for metabolomic profiling. Umbilical venous and arterial blood samples (1 mL each) were collected from a double‐clamped cord within 1 min of delivery into heparinized blood gas syringes, sealed immediately, and analyzed within 5 min using the i‐STAT System (Abbott Laboratories, Chicago, IL) for measurements of pO_2_, pCO_2_, and pH.

Umbilical venous blood was also collected into (a) an EDTA tube (3 mL) for plasma metabolomics and (b) a BD Vacutainer CPT Cell Preparation tube (4 mL) for the isolation of cord blood mononuclear cells (CBMC). All plasma and CBMC samples were stored at −80°C until analysis.

Placental biopsies were obtained within 10 min of delivery following established protocols as in our prior work [33, 34]. After removing the basal plate, ~50 mg of villous tissue was sampled midway between the cord insertion and placental margin, rinsed in ice‐cold phosphate‐buffered saline, snap‐frozen in liquid nitrogen, and stored at −80°C for downstream metabolomic and protein analyses.

### 2.3 Placental and Plasma Metabolomics

Placental and plasma metabolomics were conducted in a single batch. Placental profiles (*n* = 50; 28 preeclampsia cases) were used for haplotype‐phenotype association analyses based on genotyping data availability. For a subset of 28 maternal–infant pairs (14 preeclamptic), metabolomic data were collected from paired maternal plasma, placental tissue, and umbilical venous plasma to assess (a) consistency of metabolite profiles across compartments, and (b) whether preeclampsia‐associated alterations were shared across the maternal–placental–fetal axis.

Samples were prepared for ultra‐high‐pressure liquid chromatography (UHPLC)‐mass spectrometry metabolomics as described [35]. Placental tissues were diluted to 15 mg/mL. UHPLC–MS was performed on a Vanquish UHPLC system coupled with a high‐resolution Q Exactive mass spectrometer (Thermo Fisher, Breman, Germany). Samples were randomized and analyzed in positive and negative ionization modes (10 μL injection volume). Solvent phases were water (a) and acetonitrile (b) supplemented with 0.1% formic acid (positive mode) or 1 mM ammonium acetate (negative mode). Metabolites were separated on a Kinetex C18 column (2.1 × 150 mm, 1.7 μm) using a 5‐min gradient [35].

Raw data files were converted to mzXML format using RawConverter [36]. Metabolites were identified using Maven (Princeton, NJ, USA) and annotated via the KEGG database [37]. Peak intensities were normalized in MetaboAnalyst 6.0 [38] using Pareto scaling and log transformation.

### 2.4 Genotyping

Fetal genotyping and selection scan analysis on 50 samples followed our previously published protocols [15]. Genomic DNA was extracted from CBMCs using the AllPrep DNA/RNA Mini Kit (Qiagen); only samples with a DNA Integrity Number > 8 were included. Genotyping was performed using the Illumina Multiethnic Genotyping Array (MEGA, 1.8 million SNPs) [39] at the Colorado Center for Personalized Medicine. SNPs with a call rate < 95%, low clustering quality, or violating Hardy–Weinberg Equilibrium (*p* < 0.001 after population structure correction) were excluded.

### 2.5 Selection Scan Analysis

MEGA‐derived genotypes were converted to variant call format (VCF) and quality‐filtered using VCFtools v0.1.16. Filtered VCF files were mapped to the *Homo sapiens* hg19 reference genome and phased using SHAPEIT v4.2.2 with default settings (seven total burn‐in iterations, three total pruning iterations, and five main iterations). The integrated haplotype score (iHS) was calculated using the rehh R package v3.2.2 [40]. The iHS statistic [41] is a robust and widely validated method for detecting recent selective sweeps arising from de novo mutations or standing genetic variation, while minimizing confounding from background selection [42, 43]. It has undergone extensive benchmarking [41, 44] and has been successfully applied to identify adaptive signals in high‐altitude populations [15]. An absolute iHS of three or greater (|iHS| ≥ 3) was considered strong evidence of positive natural selection. Each selected haplotype was defined by the top three variants within the haploblock containing the highest iHS signal. Participants were assigned a genotype score of 0, 1, or 2 based on the copy number of these putatively adaptive haplotypes.

### 2.6 Mitochondrial Unfolded Protein Tissue Lysate Preparation and Western Blot

While the mitochondrial unfolded protein response (UPR^mt^) expression data and methods have been published previously [15], the relationship of these protein markers to fetal haplotypes is unique to this study. We assessed the abundance of four key (UPR^mt^) proteins—TID1 (tumorous imaginal disc protein 1), CLPP (caseinolytic mitochondrial matrix peptidase proteolytic subunit), HSP60 (heat shock protein 60) and ATF5 (activating transcription factor 5).

Placental lysates were prepared at a concentration of 2.5 mg/uL in gel loading buffer (50 mM Tris–HCl, pH 6.8; 100 mM DTT; 2% SDS; 10% glycerol) and heated at 70°C for 10 min. Proteins were separated by SDS‐PAGE and transferred to nitrocellulose membranes. Membranes were blocked in 5% skimmed milk (TBS‐T) for 1 h and incubated with primary antibody overnight at 4°C, followed by room temperature incubation. Primary antibodies were diluted in TBS‐T as follows: TID1 (1:1000; GeneTex, GTX111077), CLPP (1:000, Abcam, ab124822), HSP60 (1:4000; Abcam, ab46798), ATF5 (1:1000; Abcam, ab184923). HRP‐conjugated secondary antibodies (GE Healthcare, UK) were applied for 1 h. Detection was performed via enhanced chemiluminescence (Amersham Biosciences, UK) and band intensities were quantified using Image J (NIH). All protein levels are expressed relative to β‐actin.

### 2.7 Statistical Analysis

After evaluating data normality using the Kolmogorov–Smirnov test, maternal and newborn characteristics were contrasted between normotensive controls and preeclamptic cases using unpaired Student's *t*‐test or Chi‐square test, as appropriate. These included comparisons of clinical characteristics and normalized peak intensities for prioritized metabolites (L‐carnitine, acylcarnitines and fatty acids). Associations between continuous variables (e.g., metabolite abundance and birthweight) were evaluated via simple linear regression. Estimated marginal birthweight means were generated and compared using univariate general linear models, adjusting for gestational age and infant sex. For these analyses, statistical significance was set at *p* ≤ 0.05. Data analyses were conducted using SPSS v.28 (IBM, Armonk, NY) or GraphPad Prism v. 8.4.3 (GraphPad Software Inc., La Jolla, CA).

Multivariate analyses on metabolomic profiles were performed using MetaboAnalyst v6.0 [38]. For each compartment, metabolite peak intensities were Pareto scaled and log transformed (base 10). A low variance filter was applied (interquartile range, 5%) to exclude near‐constant features across preeclampsia‐control [45]. Normalized metabolite peak intensity data were subjected to orthogonal partial least squares discriminant analysis (OPLS‐DA) to assess separation between control and preeclamptic metabolomic profiles [46]. Discriminant metabolites were defined as those lying ±1.5 SD from the model mean. Differences in discriminant metabolite abundances were tested using Student's *t*‐tests, followed by false discovery rate (FDR) correction via the two‐stage linear step‐up procedure of Benjamini, Krieger, and Yekutieli, *Q* = 1%.

Associations between individual metabolites and birthweight were evaluated using raw peak intensity values and simple linear regression with *p* ≤ 0.05 considered significant. Haplotype‐metabolite associations were tested using putatively adaptive haplotypes (|iHS| ≥ 3) and raw peak intensity data. Haplotypes were included only if at least two haplotype copy number groups (0,1, or 2) had more than 4 subjects. Linear regression models were used, with a more stringent threshold of *p* ≤ 0.01 to improve power and reduce false positives.

## 3 Results

Maternal and newborn characteristics are shown in Table 1. Preeclamptic and normotensive women were of similar age, height, prepregnant weight and body mass index, gravidity, parity, and all were delivered by unlabored scheduled caesarean section. In both groups, the average maternal altitude of birth and childhood exceeded 3000 m, and all women had lived at high altitudes for most of their lives. Hemoglobin concentrations were 1.8 g/dL higher in preeclamptic than in normotensive women, while resting oxygen saturation did not differ between groups. Compared to normotensive controls, preeclamptic patients had a higher incidence of preterm births (59%), with 42% being born small for gestational age and average birth weights reduced by 572 g after adjusting for gestational age and sex.

**TABLE 1:** Maternal and newborn characteristics.

Variables	Control	Preeclampsia	*p*
**A. Maternal characteristics**			
Maternal age, years	32.7 ± 5.4	33.5 ± 5.3	0.53
Altitude of birth, m	3427 ± 868	3969 ± 253	0.10
Altitude of childhood, m	3457 ± 902	3771 ± 259	0.07
Duration HA residence, years	30.5 ± 8.1	32.1 ± 6.2	0.41
Weight (prepregnant), kg	61.2 ± 11.4	63.3 ± 9.7	0.48
Height, m	1.55 ± 0.07	1.54 ± 0.06	0.75
Body mass index, kg/m^2^	25.6 ± 4.1	26.6 ± 4.1	0.37
SpO_2_, %	92.6 ± 3.9	89.5 ± 17.4	0.30
Hemoglobin, g/dL	14.4 ± 2.1	16.2 ± 2.2	0.001
Gravidity	2.1 ± 0.7	2.2 ± 1.1	0.63
Parity	0.97 ± 0.68	0.90 ± 0.92	0.73
Prenatal visits, #	5.6 ± 1.7	4.8 ± 2.0	0.15
First prenatal visit, week	19.9 ± 9.0	17.8 ± 6.6	0.36
**B. Newborn characteristics**			
GA, weeks	37.9 ± 2.2	35.1 ± 3.3	< 0.001
Preterm, %	17 [7, 34]	59 [41, 75]	< 0.001
Birth weight (BW), g	3158 ± 537	2085 ± 799	< 0.001
BW* (adjusted), g	2948 ± 93	2376 ± 94	< 0.001
BW below 5th percentile %	3.6 [0.2, 17.7]	42.3 [25.5, 61.1]	< 0.001
BW below 10th percentile %	7.1 [1.3, 22.6]	42.3 [25.5, 61.1]	0.002
Infant sex (male), %	58 [40, 74]	64 [45, 79]	0.66
Apgar (5‐min)	8.9 ± 0.3	8.6 ± 0.7	0.02

*Note:* Shown are values for the full cohort, *n* = 33 control, *n* = 30 preeclampsia, presented as means ± standard deviation for continuous variables or percentage and 95% CI for proportions. BW* Adjusted for gestational age at delivery and infant sex (for this variable only the estimated marginal mean and SEM are shown).

Abbreviations: BW, birthweight; GA, gestational age; HA, high altitude; SpO_2_, peripheral oxygen saturation.

### 3.1 Distinct Acylcarnitine and Fatty Acid Metabolite Profiles in Preeclampsia

**FIGURE 1:**
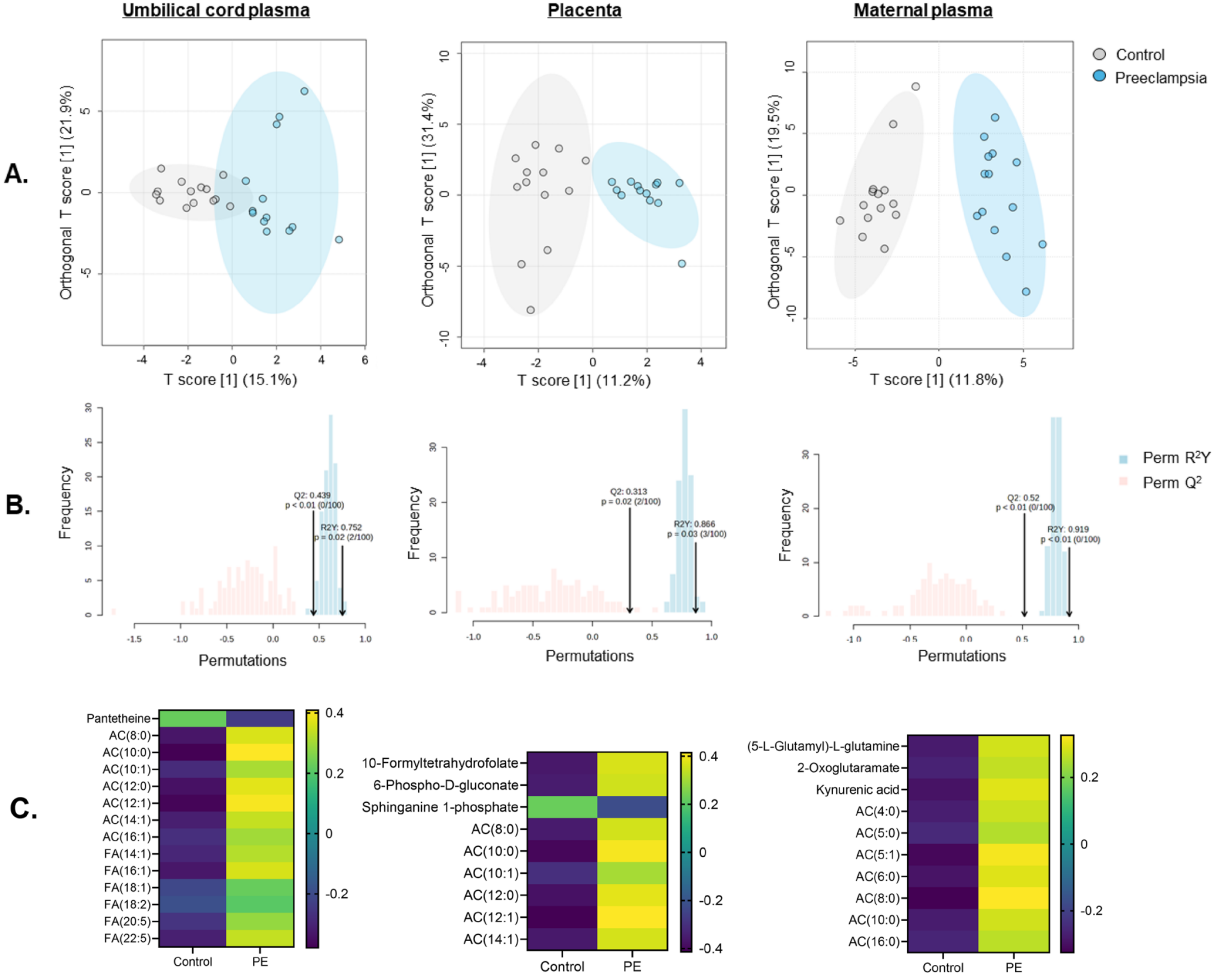
Differential metabolomic profiles in preeclampsia (PE) across maternal and fetal compartments. (A) Analysis of metabolomic profiles across cord plasma (left), placenta (center), and maternal plasma (right) using orthogonal partial least squares discriminant analysis (OPLS‐DA) in control (CON, white) and preeclampsia (PE, blue). Each OPLS‐DA model was validated using a permutation (100) test and presented as permR^2^ and permQ^2^ values (B). Before analysis, values were normalized using pareto scaling and log transformation. Discriminant metabolites were identified from an S plot as ±1.5 SD from the mean. (C) The control vs. preeclampsia metabolite values for top model discriminants were tested using a T‐test followed by a false discovery rate correction two‐stage linear step‐up procedure of Benjamini, Krieger, and Yekutieli, *Q* = 1%. Significant values from this are presented in the heatmap as normalized values. *N* = 14 control, 14 preeclampsia (PE).

Using MetaboAnalyst 6.0, we employed OPLS‐DA [46] to test the separation of case–control (preeclampsia‐normotensive) metabolomic profiles across umbilical venous plasma, placenta, and maternal plasma (Figure 1A). Significant separation of preeclampsia and normotensive cases was confirmed through a permutation test (100 iterations), with permQ^2^ values with *p* < 0.01 and permR^2^ values with *p* < 0.05 across all compartments (Figure 1B). Discriminant metabolite analysis showed elevated acylcarnitine abundance in preeclamptic cases in all compartments (Figure 1C). In umbilical cord plasma, both acylcarnitines and fatty acids were greater in preeclampsia than in controls.

**FIGURE 2:**
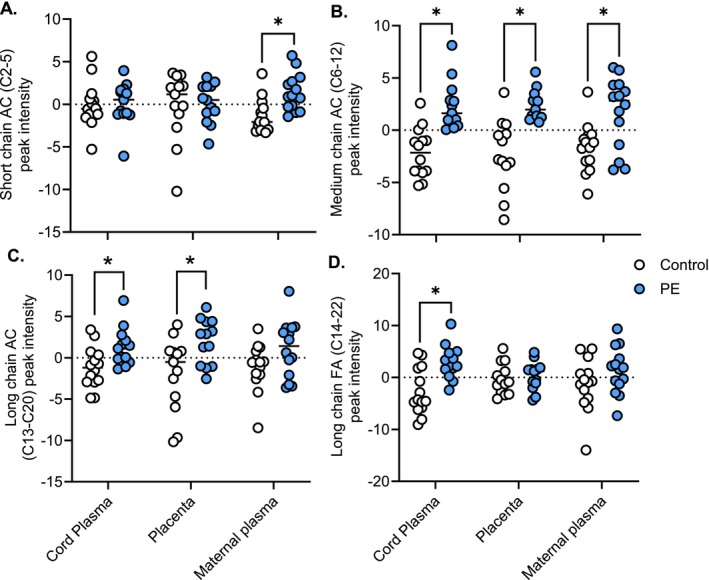
Abundance of acylcarnitines (AC) and fatty acids (FA) in umbilical cord plasma, placenta, and maternal plasma in normotensive (control) and preeclamptic (PE) pregnancy. (A) Short‐chain AC, carbon chain lengths (C) 2–5 were raised in maternal plasma in PE. (B) Medium‐chain AC (C6‐12), were raised across cord plasma, placenta, and maternal plasma in PE. (C) Long‐chain AC (C13‐20) were raised in cord plasma and placenta in PE. (D) Long‐chain fatty FAs (C14‐22) were raised in cord plasma in PE. Peak intensities were normalized using Pareto scaling and log transformation. Control and preeclamptic values were compared using an unpaired Student's t‐test. Significant differences in metabolite abundance between control and PE pregnancies are indicated by an asterisk, *p < 0.05. *n* = 14 control (white), 14 preeclampsia (PE, blue).

Given their functional significance in fatty acid transport and oxidation, we examined acylcarnitines by carbon chain length (short‐chain, medium‐chain, or long‐chain). In maternal plasma, short‐chain acylcarnitines (C2–5) were 75% higher in preeclampsia (Figure 2A). Medium‐chain (C6–12) acylcarnitines were consistently elevated across compartments: by 120% in cord plasma, 86% in placenta, and 98% in maternal plasma (Figure 2B). Long‐chain (C13–20) acylcarnitine abundance was also increased in preeclampsia, by 39% in cord plasma and 76% in placenta (Figure 2C). Free carnitine (L‐carnitine) levels were unchanged (Figure S1A). Long‐chain fatty acids in cord plasma were 22% higher in preeclampsia cases (Figure 2D), while medium‐chain fatty acid levels did not differ (Figure S1B). When stratified by saturation, monounsaturated (MUFA) and polyunsaturated fatty acids (PUFA) were elevated by 68% and 41%, respectively, in preeclampsia, with no change in the MUFA/PUFA ratio (Figure S2A–C).

**FIGURE 3:**
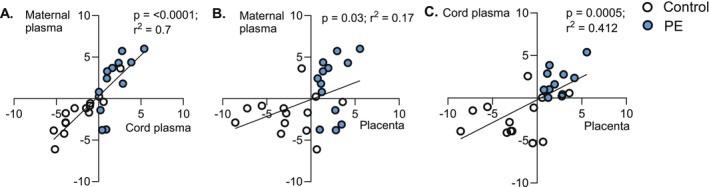
Relationship of medium‐chain acylcarnitine abundance across compartments within individuals. Significant within‐subjects associations were observed between maternal plasma and cord plasma (A), maternal plasma and placenta (B), and cord plasma and placenta (C). Statistical analysis was conducted using simple linear regression adjusted to gestational age. *n* = 14 controls (white), *n* = 13 preeclamptic (PE; blue).

Given the consistent elevation of medium‐chain acylcarnitines across compartments in preeclampsia, we tested for intra‐subject concordance. Acylcarnitine levels were strongly correlated across the maternal–placental–fetal axis, independent of preeclamptic status (Figure 3).

### 3.2 Acylcarnitine Accumulation Associates With Low Birthweight

**FIGURE 4:**
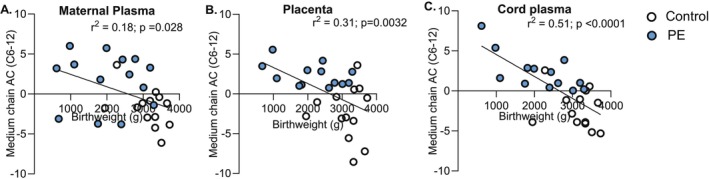
Relationship between medium‐chain acylcarnitine (AC) abundance and birth weight. Birthweight was inversely associated with medium chain AC levels in maternal plasma (A), placenta (B), and cord plasma (C). Statistical analysis was conducted using simple linear regression adjusted to gestational age. *n* = 13 controls (white), *n* = 14 PE (blue).

To assess whether medium‐chain acylcarnitine accumulation is linked to fetal growth, we tested for associations between metabolite abundance and birthweight across maternal, placental, and fetal compartments. Elevated medium‐chain acylcarnitines were consistently associated with lower birthweight in normotensive and preeclamptic subjects (Figure 4).

Broadly, several acylcarnitine species were inversely associated with birthweight (Table S1). Among short‐chain acylcarnitines, acetyl‐carnitine (C2) and butyryl‐carnitine (C4) were significantly associated with lower birthweight in maternal and cord plasma, but not in the placenta. Medium‐chain acylcarnitine species, including hexanoyl‐ (C6), octanoyl‐ (C8) and decanoyl‐carnitine (C10), were significantly associated with low birthweight in all compartments, most consistently in cord plasma. Long‐chain species (C14–18) also showed significant inverse associations in cord plasma and placenta, with arachidonoyl carnitine (C20:4) associated in placenta only. Increased L‐carnitine abundance was also inversely associated with birthweight in cord and maternal plasma (Table S1).

For fatty acids, the medium‐chain species octanoic acid (C8) and decanoic acid (C10) were negatively associated with birthweight in cord plasma (Table S2).

### 3.3 Adaptive Fetal Haplotypes Associate With Placental Metabolite Abundance

**FIGURE 5:**
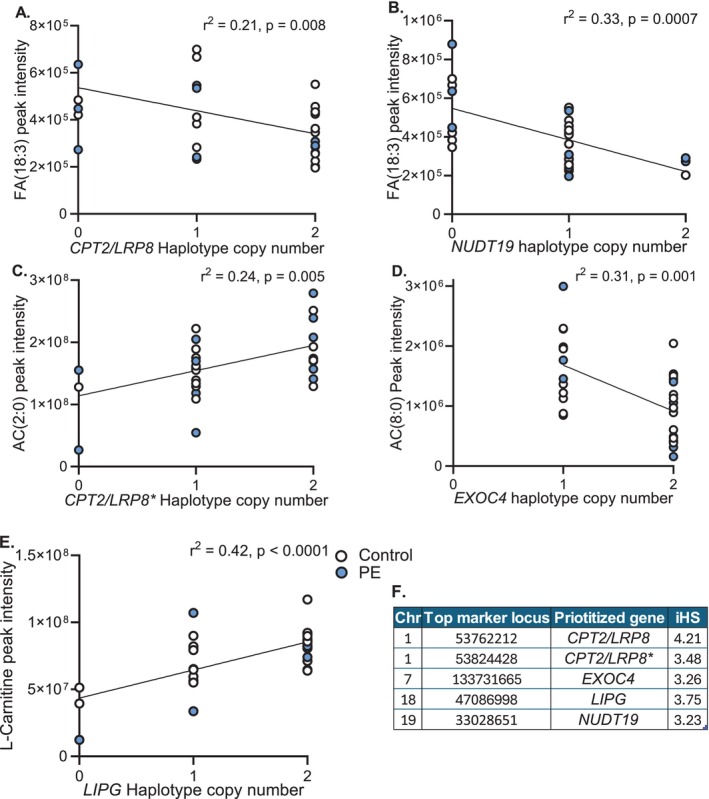
Genotype–phenotype association analyses reveal novel links between adaptive fetal haplotypes and the abundance of placental metabolites. Scatterplots illustrate the relationship between putatively adaptive haplotype copy number (0, 1, 2) and placental metabolite abundance. The associated placental metabolites included: Fatty acids (FA) carbon chain lengths 18:3 (A, B), acylcarnitines (AC) carbon chain lengths 2:0 and 8:0 (C, D), L‐carnitine (E). Linear regression analyses identified associations; *p* value < 0.01 was considered significant. *n* = 28 control (white), *n* = 22 preeclampsia (PE, blue). (F) Table detailing the fetal haplotype location, including chromosome (Chr) and top marker position (hg19 reference genome), the gene within the 200 kb haplotype region most functionally relevant to lipid metabolism (prioritized gene), and the iHS score. Abbreviations: Carnitine palmitoyl transferase 2 (*CPT2*), low density lipoprotein receptor‐related protein 8 (*LRP8*), exocyst complex component 4 (*EXOC4*), lipase G endothelial type (*LIPG*), nudix hydrolase 19 (*NUDT19*). An asterisk distinguishes haplotypes containing the same prioritized genes (*CPT2/LRP8*) *.

To examine whether placental metabolic profiles distinguishing normotensive and preeclamptic pregnancies reflect adaptive processes, we tested for associations between placental metabolite abundance (fatty acids, acylcarnitines and L‐carnitine) and adaptive fetal haplotypes (|iHS| ≥ 3). Genes within 200 kb of the top tagging SNPs were annotated, and those with metabolic functions, particularly in lipid metabolism, were prioritized (Figure 5).

Several associations emerged. Individuals carrying adaptive haplotypes annotated to carnitine palmitoyl transferase 2 (*CPT2*) and low‐density lipoprotein receptor‐related protein 8 (*LRP8*) had reduced placental linolenic acid (18:3) abundance (Figure 5A). A similar reduction was observed in carriers of haplotypes near *NUDT19* (Figure 5B). Conversely, carriers of the *CPT2/LRP8* haplotype showed elevated levels of short‐chain acetyl‐carnitine (C2:0) (Figure 5C), while medium‐chain acylcarnitine (C8:0) was decreased in individuals with a haplotype containing exocyst complex component 4 (*EXOC4*) (Figure 5D). Placental L‐carnitine was significantly elevated in carriers of adaptive haplotypes containing Lipase G endothelial type (*LIPG*) (Figure 5E). A summary of these haplotypes, including iHS scores and prioritized genes, is provided in Figure 5F, with all significant genotype–metabolite associations listed in Table S3.

### 3.4 Adaptive Placental Metabolic Signals and Mitochondrial Stress Responses

**FIGURE 6:**
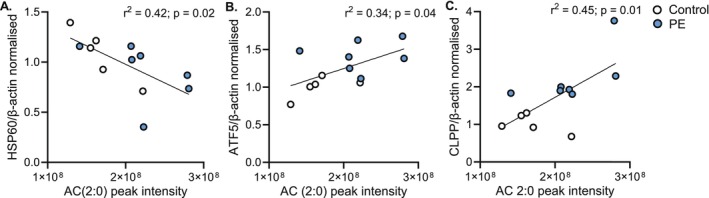
Adaptive placental metabolic signals associate with the expression of mitochondrial unfolded protein response proteins. Acylcarnitine (AC) 2:0 Abundance was negatively associated with (A) heat shock protein 60 (HSP60) and positively associated with (B) activating transcription factor 5 (ATF‐5) and (C) caseinolytic peptidase P (CLPP). Placental protein expression was normalized to β‐Actin. Statistical analysis was performed using simple linear regression. A‐C, *n* = 5 control (white), 7 preeclampsia (PE, blue).

To investigate the potential functional relevance of adaptive metabolic signals in the placenta, we examined associations between metabolite abundance and markers of mitochondrial function and oxidative stress, using previously published data from this cohort [15]. Placental acetyl‐carnitine (C2:0) levels were significantly associated with the expression of (UPR^mt^) components, indicating a link between altered lipid metabolism and mitochondrial proteostatic stress. This includes a negative association with heat shock protein 60 (HSP60) (Figure 6A), a molecular chaperone that exhibits a variety of functions, typically operating within the mitochondria to facilitate the folding of nascent proteins [47, 48]. Placental acetyl‐carnitine levels demonstrated positive associations with activating transcription factor 5 (ATF‐5) (Figure 6B), a transcriptional regulator of UPR^mt^ component expression [49], and caseinolytic peptidase proteolytic subunit (CLPP) (Figure 6C), a quality control mitochondrial protease that recognizes and degrades misfolded proteins [50, 51].

## 4 Discussion

Our findings reveal that distinct metabolic profiles, characterized by acylcarnitine and fatty acid abundance, are associated with preeclampsia and often show opposing associations with adaptive fetal haplotypes in highland Andeans, implicating lipid metabolism as a target of natural selection in this population. Specifically, we identified an accumulation of medium‐ and long‐chain acylcarnitines across maternal, placental, and fetal compartments in preeclamptic pregnancy, consistent with impaired fatty acid oxidation compared to normotensive pregnancy. The accumulation of these acylcarnitine species was tightly linked to reduced birthweight, a critical determinant of neonatal survival and lifelong health. Fetal haplotypes with strong signals of positive selection were associated with opposing metabolite signatures. These haplotypes contain genes with well‐established roles in lipid transport and oxidation (e.g., *CPT2*, *LRP8*, *LIPG*, *NUDT19*), indicating lipid metabolism is a key target of natural selection in Andeans. Given the dual challenge of hypoxia and high energy demand during pregnancy, our findings support a genetic basis for adaptive placental phenotypes in highland Andeans, specifically indicating strong selective pressure on placental fatty acid metabolism.

Acylcarnitines are obligate intermediates for mitochondrial fatty acid oxidation, facilitating the transport of fatty acids across the mitochondrial membrane for β‐oxidation. When fatty acid oxidation is incomplete, acylcarnitines can accumulate and be exported to the circulation, making their plasma levels useful indicators of substrate flux and metabolic dysfunction. Clinically, plasma acylcarnitine levels are used to detect inborn errors of metabolism, including disorders of fatty acid catabolism in neonates [52]. In this study, we observed a consistent accumulation of medium and long‐chain acylcarnitines (C6‐C22) across the maternal plasma, umbilical cord plasma, and placenta in preeclamptic pregnancies, indicating widespread disruption to fatty acid metabolism in Andean preeclampsia cases across the maternal–placenta–fetal axis. This pattern is consistent with incomplete fatty acid oxidation, whereby a large portion of fatty acids entering the mitochondria are only partially degraded [53]. Our findings align with previous reports in lowland populations showing increased acylcarnitine abundance across different chain lengths in maternal and umbilical cord plasma in preeclampsia [54], as well as reduced expression of placental fatty acid oxidation enzymes and suppressed palmitate oxidation [20, 25]. While there is strong evidence of altered acylcarnitine profiles in maternal plasma and the placenta in preeclampsia, as well as for placental acylcarnitine transfer to the fetus, explicit triad studies measuring all three compartments have not been reported; most studies focus on two of the three compartments, with the triad relationship inferred. To our knowledge, this is the first study to directly compare acylcarnitine profiles across the maternal–placenta–fetal axis in maternal–infant pairs, providing a more integrated view of systemic metabolic dysfunction in preeclampsia, particularly in the context of high‐altitude pregnancy.

Fetal development depends on fatty acids for energy production, neurological development and cell membrane structure [55, 56]. In the umbilical vein, which carries oxygenated blood from the placenta to the fetus, we observed an increase in long‐chain fatty acids, including MUFAs and PUFAs, during preeclamptic pregnancy. While the exact source of elevated fatty acids in the fetal compartment remains uncertain, our findings align with previous reports of increased fatty acid content in umbilical vessel walls in preeclampsia [57, 58]. The fetus can synthesize limited MUFAs from glucose but cannot produce essential fatty acids or sufficient PUFAs. Therefore, it relies on the transport of fatty acids across the placenta, primarily through the hydrolysis of triglyceride‐rich lipoproteins [56]. The observed rise in fetal fatty acids may result from increased lipolytic activity, which is five times higher in maternal plasma during preeclampsia [59], in combination with impaired placental fatty acid oxidation. Indeed, placental oxidative capacity influences the availability of lipids for fetal use [60]. Therefore, increased umbilical cord fatty acids may reflect compensatory lipid transfer in the context of mitochondrial dysfunction in the placenta. In contrast, carnitine species are not actively transferred across the placenta, with minimal passive diffusion [61]. This suggests that the rise in acylcarnitines in umbilical cord plasma likely reflects fatty acid oxidation in fetal tissues. Together, these findings suggest that altered placental lipid metabolism in preeclampsia disrupts maternal‐fetal lipid partitioning and fetal mitochondrial function, metabolic vulnerabilities that may have been targeted by natural selection in highland populations.

High acylcarnitine abundance has been linked to reduced birthweight in lowland populations, both in maternal [62] and newborn plasma [63]. Consistent with these findings, we observed that elevated levels of short‐, medium‐, and long‐chain acylcarnitines across all compartments were associated with lower birth weight in our highland Andean cohort, except for short‐chain acylcarnitines in the placenta. Given that birth weight is a key indicator of maternal and fetal health [11] and a predictor of future health outcomes [10], these findings underscore the potential impact of impaired fatty acid oxidation on determining reproductive success in highland Andeans.

In contrast to these maladaptive metabolic patterns, we identified metabolite signatures linked to putatively adaptive fetal haplotypes. These relationships, in some cases, oppose those found in preeclampsia and include decreased linolenic acid (18:3), increased acetyl‐carnitine (C2) and L‐carnitine, and decreased octanoyl‐carnitine (C8). While these metabolites are involved in fatty acid oxidation, acetyl‐carnitine (C2) may also arise from amino acid metabolism [53], suggesting broader shifts in placental energy pathways. Notably, acetyl‐carnitine abundance was positively associated with the expression of proteins involved in the mitochondrial unfolded protein response, including ATF5 and CLPP, and negatively associated with HSP60, linking metabolic adaptation to an enhanced placental mitochondrial quality control system in high‐altitude pregnancy [26, 64]. This suggests that acylcarnitines may not only reflect metabolic flux but also play a role in regulating mitochondrial function under hypoxic stress during high‐altitude pregnancy.

While the direct mechanisms by which these genotypes influence placental metabolism remain to be elucidated, their proximity to genes involved in lipid metabolism points to candidate adaptive pathways. Given that mitochondrial fatty acid oxidation is a highly oxygen‐consuming process [65] and is suppressed in cardiac and skeletal muscle under hypoxia [66, 67, 68], selective pressure on lipid metabolism at high altitude is plausible. Indeed, in Tibetan Sherpa, selection on peroxisome proliferator‐activated receptor‐α (*PPARA*), a master regulator of fatty acid oxidation, has been linked to reduced skeletal muscle fatty acid oxidation and improved oxygen utilization efficiency [1]. Our findings suggest that similar selective forces may act on placental metabolism in Andean highlanders.

We identified positive selection signals in several fetal haplotypes associated with placental lipid metabolism, suggesting coordinated selection of key metabolic pathways in highland Andeans. Among these were two haplotypes encompassing *CPT2* and *LRP8*, genes previously shown to be under selection in Peruvian highlanders [69]. Both genes are highly expressed in the placenta [70, 71], and genetic variation in *LRP8* has been associated with improved reproductive outcomes, including higher birth weight and lower FGR risk in lowland populations [72]. In this study, we report novel associations between the *CPT2/LRP8* haplotypes and placental abundance of linolenic acid and acetyl‐carnitine, both key components of fatty acid metabolism. *LRP8* encodes apolipoprotein E (ApoE)‐receptor 2, which facilitates cellular uptake of ApoE‐containing lipoproteins central to lipid transport and clearance through both lipolysis and hepatic VLDL production [70]. *CPT2* encodes carnitine palmitoyltransferase 2, which catalyzes the final step of transporting long‐chain acylcarnitines into the mitochondrial matrix for ꞵ‐oxidation. Adaptive variation in the *CPT2/LRP8* haplotypes likely influences lipid transport and utilization, potentially working together to downregulate long‐chain fatty acid oxidation.

A putatively adaptive fetal haplotype containing *NUDT19*, which encodes Nudix Hydrolase 19, was associated with reduced placental linolenic acid abundance. NUDT19 regulates intracellular acyl‐CoA levels through hydrolysis [73], and has been implicated in the control of lipid metabolism and mitochondrial function in hepatocytes by modulating CoA availability [74]. It is also a component of the mitochondrial matrix in the human placenta [75]. Given the central role of acyl‐CoA in fatty acid synthesis and oxidation, we hypothesize that NUDT19 contributes to placental adaptation by modulating long‐chain fatty acid flux under hypoxic stress. We report a novel association between a putatively adaptive fetal haplotype containing *NUDT19* and decreased placental linolenic acid abundance. We postulate this association is linked to the role of NUDT19 in regulating long‐chain fatty acid oxidation by modulating fatty acyl‐CoA availability.

Similarly, we identified an adaptive fetal haplotype containing *LIPG*, which encodes endothelial lipase, a key enzyme involved in hydrolyzing triglycerides and phospholipids to facilitate fatty acid uptake. *LIPG* has been implicated in metabolic disorders, for instance, being identified as a pro‐atherogenic factor in metabolic syndrome [76]. In the placenta, *LIPG* expression is diminished in obesity [77], suggesting sensitivity to maternal metabolic status. Here, we present a novel association between *LIPG* and increased placental L‐carnitine, which may reflect a role for *LIPG* in regulating placental fatty acid oxidation.

Finally, an adaptive haplotype region encompassing *EXOC4* was associated with decreased placental octanoyl‐carnitine, contrasting with the accumulation observed in preeclamptic pregnancies. EXOC4, an exocyst complex component, facilitates insulin‐stimulated GLUT4 translocation [78] and regulates adipocyte fatty acid uptake [79]. Although exocyst proteins, including EXOC4, are expressed in the human placenta [80], their role in placental metabolism and lipid trafficking remains largely unexplored. Our findings suggest that selection has acted on multiple components of placental fatty acid handling, potentially enhancing mitochondrial efficiency and lipid homeostasis to support reproductive success in chronically hypoxic environments.

The genotype–phenotype associations we identified in highland Andeans are novel and may represent unique modifications to placental lipid homeostasis and broader metabolic function supporting reproductive success under hypoxic conditions. Beyond direct metabolic effects, future work should also consider the impact of these genotype–phenotype associations on other physiological systems that are important for human adaptation to high altitude and successful pregnancy outcomes across altitude gradients. For example, lowland ancestry women living at moderate altitudes in Colorado with higher plasma acylcarnitine abundance have lower uterine artery blood flow at 34 weeks of pregnancy, a phenotype consistently linked to reduced fetal growth [81]. Acylcarnitine exposure also suppresses human umbilical vein endothelial cell angiogenesis [82, 83], suggesting multiple avenues by which lipid metabolism may impact pregnancy outcome.

Our study has several strengths that advance understanding of placental metabolic function in highland Andean pregnancy within an evolutionary context. First, we focused on Andeans in Bolivia—an understudied population living at high altitude, where chronic hypoxia and disproportionally high rates of preeclampsia pose significant maternal‐fetal health challenges [84, 85]. With half the population living above 3000 m and the highest under‐5 mortality rate in Latin America, this setting highlights an urgent public health need [86]. Children born to mothers with preeclampsia also have a higher risk of developing neonatal pulmonary hypertension [16] and elevated BP in adulthood [87]. High‐altitude preeclampsia, driven by a more uniform stressor (hypoxia), provides a more controlled environment for isolating the interaction of genetics and effects of oxygen limitation on pregnancy. Second, we used a triad‐based design—profiling metabolites across matched maternal plasma, placenta, and cord plasma—providing rare insight into metabolic coordination across the maternal–placental–fetal axis and, to our knowledge, the first such analysis in any lowland or highland population. Third, by integrating metabolomics with fetal genomic data, we identified maladaptive and potentially adaptive metabolic signatures, thereby distinguishing between pathology and evolutionarily beneficial responses. Finally, we uncovered novel associations between placental metabolite levels and fetal haplotypes in genes related to lipid transport, fatty acid oxidation, and mitochondrial function. These findings provide mechanistic insight into how natural selection may shape placental metabolism under chronic hypoxia.

While our study advances the understanding of the metabolic origins of preeclampsia at high altitude and the role of adaptive genetic variants in regulating placental metabolism in Andean highlanders, we acknowledge its limitations. The modest sample size limited the application of genome‐wide association analysis, underscoring the need for larger cohorts to facilitate the discovery and validation of risk variants and adaptive loci. In addition, next‐generation sequencing of the candidate regions identified here is required to pinpoint functional variants of interest. Although strong correlations were observed between key metabolites and adaptive haplotypes, definitive mechanistic links remain unresolved. Functional experiments using placental explants, CRISPR‐mediated allele editing or in vivo models such as transgenic mice with adaptive genetic variants will be essential to evaluate causal effects of genetic variation on placental metabolic pathways. Finally, expanding the range of lipid classes through lipidomics could uncover additional pathways involved in adaptation or dysfunction.

Our findings lay a foundation for improving maternal–infant health by identifying distinct acylcarnitine and fatty acid signatures associated with preeclampsia, paving the way for biomarker development and early risk screening in high‐altitude pregnancies. Furthermore, by linking genetic haplotypes to beneficial placental metabolic phenotypes, this study supports the prospect of risk stratification and individually tailored prenatal care based on genetic background. Finally, implicating mitochondrial fatty acid oxidation as a critical adaptive pathway in preeclampsia, our results highlight a novel candidate therapeutic avenue.

In summary, our findings reveal adaptive mechanisms related to placental lipid homeostasis, which may be central to reproductive success in Andean highlanders. By integrating metabolomic and genomic data, we identified disrupted fatty acid oxidation as a hallmark of preeclampsia across the maternal‐placental‐fetal triad and uncovered adaptive fetal haplotypes associated with distinct placental metabolic profiles. These results suggest evolutionary processes supporting reproductive success under chronic hypoxia by modulating placental energy metabolism. Our study not only provides novel insights into the metabolic mechanisms behind pregnancy complications at high altitude but also emphasizes the importance of integrating evolutionary and systems biology approaches to understand human adaptation and disease.

## Author Contributions

C.G.J. designed research; L.T.‐J., L.L.‐V., V.M.‐G. recruited subjects and collected samples; K.A.O. and C.G.J. analyzed data; W.G., J.A.H., H.W.Y., H.Y., J.A.R., J.S., M.S., A.D. performed research; L.G.M. and T.S.S. reviewed and contributed to the paper, K.A.O. and C.G.J. wrote the paper.

## Conflicts of Interest

The authors declare no conflicts of interest.

## Supporting information


**Figure S1:** Abundance of L‐carnitine or medium‐chain fatty in normotensive (control) or preeclamptic (PE) pregnancy.

**Figure S2:** Comparison of fatty acid abundance in the umbilical cord venous plasma, separated by mono‐ or poly‐ unsaturated status.

**Table S1:** Association analysis between acylcarnitines across all chain lengths in the cord plasma, placenta and maternal plasma, and birthweight.

**Table S2:** Association analysis between fatty acids across all chain lengths in the cord plasma and birthweight.

**Table S3:** Fetal haplotypes associated with placental acylcarnitines, L‐carnitine and fatty acids.

**Appendix S1:** fsb271254‐sup‐0006‐supinfo.docx.

## Data Availability

Deidentified human subject data for subjects consenting to future data use will be made available on the University of Colorado repository upon acceptance of the manuscript.
