# Supplementary figures and images for: Dysregulated Fatty Acid Metabolism in Preeclampsia Among Highland Andeans: Insights Into Adaptive and Maladaptive Placental Metabolic Phenotypes
Source: FASEB J. 2025 Nov 22;39(22):e71254. doi: 10.1096/fj.202502590R (PMC12639537; doi:10.1096/fj.202502590R)

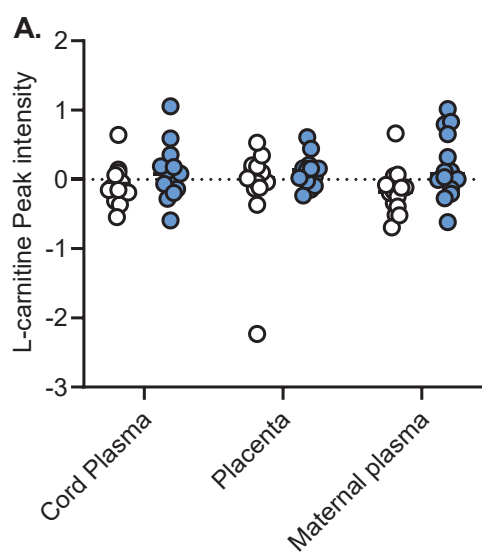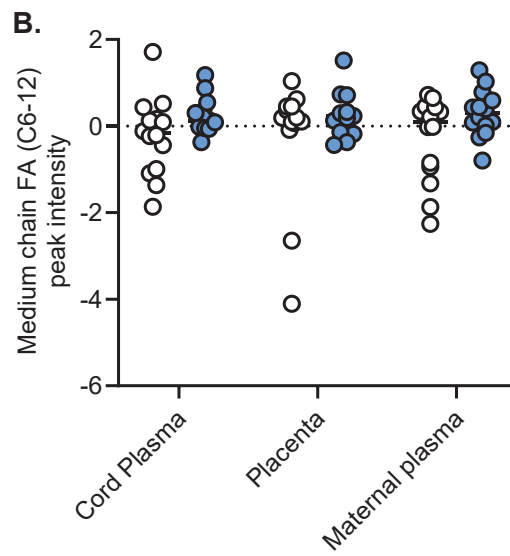

Supplement: Supplementary file 1 — Figure S1: Abundance of L‐carnitine or medium‐chain fatty in normotensive (control) or preeclamptic (PE) pregnancy. [file FSB2-39-e71254-s002.pdf]

**A. Umbilical cord plasma**

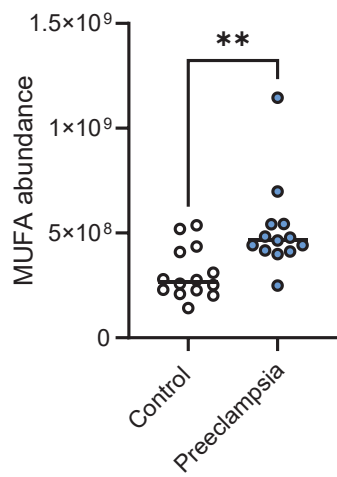

**B. Umbilical cord plasma**

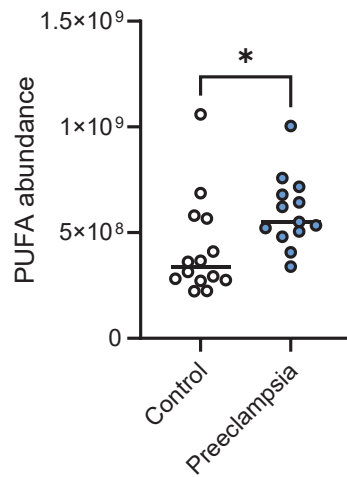

**C. Umbilical cord plasma**

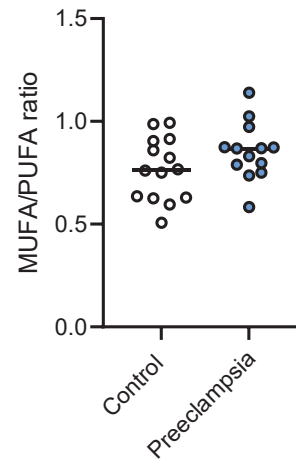

Supplement: Supplementary file 2 — Figure S2: Comparison of fatty acid abundance in the umbilical cord venous plasma, separated by mono‐ or poly‐ unsaturated status. [file FSB2-39-e71254-s003.pdf]
