# Supplementary material for: Dysregulated Fatty Acid Metabolism in Preeclampsia Among Highland Andeans: Insights Into Adaptive and Maladaptive Placental Metabolic Phenotypes
Source: FASEB J. 2025 Nov 22;39(22):e71254. doi: 10.1096/fj.202502590R (PMC12639537; doi:10.1096/fj.202502590R)
Supplement: Supplementary file 3 — Table S1: Association analysis between acylcarnitines across all chain lengths in the cord plasma, placenta and maternal plasma, and birthweight. [file FSB2-39-e71254-s006.docx]

|  | **Cord plasma** | | | | **Placenta** | | | | **Maternal plasma** | | | |
| --- | --- | --- | --- | --- | --- | --- | --- | --- | --- | --- | --- | --- |
|  | p value | R^2^ | Slope (β) | SE Slope | p value | R^2^ | Slope (β) | SE Slope | p value | R^2^ | Slope (β) | SE Slope |
| AC(2:0) | 0.0028* | 0.32 | -32622 | 9776 | 0.0381 | 0.17 | -39467 | 17985 | 0.0009* | 0.36 | -28186 | 7459 |
| AC(3:0) |  |  |  |  |  |  |  |  | 0.0028* | 0.30 | -2052 | 620.2 |
| AC(4:0) | 0.0011* | 0.36 | -232 | 62.5 |  |  |  |  | 0.001* | 0.36 | -286.5 | 77.01 |
| AC(4-OH) |  |  |  |  |  |  |  |  |  |  |  |  |
| AC(4-DC) |  |  |  |  |  |  |  |  |  |  |  |  |
| AC(5:0) |  |  |  |  |  |  |  |  | 0.0001* | 0.55 | -854.6 | 154.8 |
| AC(5:1) |  |  |  |  |  |  |  |  | 0.0003* | 0.41 | -112.5 | 27.04 |
| AC(5-OH) |  |  |  |  |  |  |  |  | 0.0001* | 0.49 | -161.5 | 33.68 |
| AC(6:0) | 0.0002* | 0.45 | -1233 | 278.1 | 0.009 | 0.25 | -4011 | 1412 | 0.0029 | 0.30 | -394.5 | 119.3 |
| AC(8:0) | 0.0001* | 0.57 | -1182 | 211.4 | 0.0178 | 0.21 | -917.7 | 360.7 | 0.0161 | 0.21 | -1111 | 430.1 |
| AC(8:1) | 0.0011* | 0.36 | -869 | 234.3 |  |  |  |  |  |  |  |  |
| AC(10:0) | 0.0001* | 0.50 | -1907 | 392 | 0.014* | 0.23 | -692.4 | 261.3 | 0.0205 | 0.20 | -1738 | 702.7 |
| AC(10:1) | 0.0007 | 0.39 | -1123 | 288.9 |  |  |  |  | 0.0086 | 0.25 | -1637 | 574.4 |
| AC(12:0) | 0.0001* | 0.46 | -1542 | 340.4 | 0.0008* | 0.38 | -1796 | 467.2 |  |  |  |  |
| AC(12:1) | 0.0002* | 0.45 | -1238 | 278.5 | 0.0029* | 0.31 | -494.3 | 149.3 |  |  |  |  |
| AC(14:0) | 0.0001* | 0.55 | -932.9 | 173 | 0.0083* | 0.26 | -5613 | 1951 |  |  |  |  |
| AC(14:1) | 0.0002* | 0.45 | -1928 | 435.6 | 0.0015* | 0.35 | -2426 | 675 |  |  |  |  |
| AC(16:0) | 0.0205 | 0.20 | -777 | 313.1 | 0.0086* | 0.25 | -19305 | 6749 | 0.0006* | 0.38 | -721.7 | 183.2 |
| AC(16:1) | 0.0001* | 0.52 | -888.8 | 173.5 | 0.0088* | 0.25 | -6380 | 2237 |  |  |  |  |
| AC(18:0) | 0.0497* | 0.15 | -116.2 | 56.23 |  |  |  |  | 0.0091* | 0.24 | -59.29 | 20.97 |
| AC(18:1) | 0.0014* | 0.35 | -392.8 | 109.2 | 0.0252* | 0.19 | -9365 | 3923 | 0.0119 | 0.23 | -526.8 | 194.1 |
| AC(18:2) |  |  |  |  |  |  |  |  | 0.0176 | 0.21 | -795.9 | 313.1 |
| AC(18:2-OH) |  |  |  |  | 0.0332* | 0.18 | -968 | 428.4 |  |  |  |  |
| AC(20:4) |  |  |  |  | 0.0084* | 0.26 | -6519 | 2271 |  |  |  |  |
| L-carnitine | 0.0044 | 0.29 | -12089 | 3842 |  |  |  |  | 0.0001* | 0.59 | -34146 | 5704 |

**Supplementary Table 1**
