# Supplementary material for: Dysregulated Fatty Acid Metabolism in Preeclampsia Among Highland Andeans: Insights Into Adaptive and Maladaptive Placental Metabolic Phenotypes
Source: FASEB J. 2025 Nov 22;39(22):e71254. doi: 10.1096/fj.202502590R (PMC12639537; doi:10.1096/fj.202502590R)
Supplement: Supplementary file 4 — Table S2: Association analysis between fatty acids across all chain lengths in the cord plasma and birthweight. [file FSB2-39-e71254-s005.docx]

|  | **Cord plasma** | | | |
| --- | --- | --- | --- | --- |
| Fatty acid (FA) | p value | R^2^ | Slope(β) | SE |
| FA(6:0) |  |  |  |  |
| FA(8:0) | 0.0026* | 0.32 | -7744 | 2299 |
| FA(9:0) |  |  |  |  |
| FA(10:0) | 0.0312 | 0.18 | -2302 | 1006 |
| FA(12:0) |  |  |  |  |
| FA(14:0) |  |  |  |  |
| FA(16:0) |  |  |  |  |
| FA(18:0) |  |  |  |  |
| FA(14:1) |  |  |  |  |
| FA(16:1) |  |  |  |  |
| FA(18:1) |  |  |  |  |
| FA(18:2) |  |  |  |  |
| FA(18:3) |  |  |  |  |
| FA(20:4) |  |  |  |  |
| FA(20:5) | 0.0207 | 0.20 | -1360 | 548.9 |
| FA(22:6) | 0.0285 | 0.18 | -10402 | 4463 |
| FA(20:3) |  |  |  |  |
| FA(22:5) |  |  |  |  |

**Supplemental Table 2**
