# Supplementary material for: Dysregulated Fatty Acid Metabolism in Preeclampsia Among Highland Andeans: Insights Into Adaptive and Maladaptive Placental Metabolic Phenotypes
Source: FASEB J. 2025 Nov 22;39(22):e71254. doi: 10.1096/fj.202502590R (PMC12639537; doi:10.1096/fj.202502590R)
Supplement: Supplementary file 5 — Table S3: Fetal haplotypes associated with placental acylcarnitines, L‐carnitine and fatty acids. [file FSB2-39-e71254-s004.docx]

**Supplemental Table 3**

| **Chr** | **Locus, top position highlighted** | **iHS** | **Associated phenotype** | **Genes in 200kb region up and downstream of top position** | **Protein encoded** |
| --- | --- | --- | --- | --- | --- |
| **1** |  |  | Fatty acid C18:3 | *SLC1A7* | Solute Carrier Family 1 Member 7 |
|  | 53750219 | 3.44 |  | ***CPT2*** | **Carnitine palmitoyl transferase 2** |
|  | **53762212** | **4.21** |  | *MAGOH* | Mago-Nashi Homolog |
|  | 53771860 | 3.98 |  | ***LRP8*** | **A low density lipoprotein receptor** |
|  |  |  |  | *CZIB* | CXXC Motif Containing Zinc Binding Protein |
|  |  |  |  | *DMRTB1* | DMRT Like Family B With Proline Rich C-Terminal 1 |
| **1** |  |  | Acetylcarnitine (C2:0) | ***CPT2*** | **Carnitine palmitoyl transferase 2** |
|  | 53820254 | 3.20 |  | *MAGOH* | Mago-Nashi Homolog |
|  | **53824428** | **3.48** |  | ***LRP8*** | **A low density lipoprotein receptor** |
|  | 53833719 | 3.17 |  | *CZIB* | CXXC Motif Containing Zinc Binding Protein |
|  |  |  |  | *DMRTB1* | DMRT Like Family B With Proline Rich C-Terminal 1 |
|  |  |  |  | *GLIS1* | GLI-related Kruppel-like zinc finger protein |
| **3** | 69743005 | 3.21 | Fatty acids C10:0, 14:1 |  |  |
|  | 69743313 | 3.21 |  | ***MITF*** | Melanocyte Inducing Transcription Factor |
|  | **69756375** | **3.25** |  |  |  |
| 6 |  |  | L-carnitine | *PRIM2* | DNA Primase Subunit 2 |
|  | **57169715** | **3.31** |  | *LINC03001* | Long Intergenic Non-Protein Coding RNA 3001 |
|  | 57182297 | 3.16 |  | *RAB23* | Member RAS Oncogene Family |
|  | 57192417 | 3.11 |  | *ZNF451* | ZNF451 Regulatory Antisense RNA 1 |
|  |  |  |  | ***BAG2*** | **BAG Cochaperone 2** |
| 7 | 133718832 | 3.05 | Acylcarnitine C8:0 | ***EXOC4*** | **Exocyst Complex Component 4** |
|  | 133726576 | 3.12 |  | *LRGUK* | Leucine Rich Repeats And Guanylate Kinase Domain Containing |
|  | **133731665** | **3.26** |  |  |  |
| 11 |  |  | L-carnitine, fatty acids C8:0, 14:1, 16:1 | *MS4A8* | Membrane Spanning 4-Domains A8 |
|  |  |  |  | *MS4A15* | Membrane Spanning 4-Domains A15 |
|  |  |  |  | *MS4A10* | Membrane Spanning 4-Domains A10 |
|  |  |  |  | *CCD86* | Coiled-Coil Domain Containing 86 |
|  |  |  |  | *PTGDR2* | Prostaglandin D2 Receptor 2 |
|  |  |  |  | *ZP1* | Zona Pellucida Glycoprotein 1 |
|  | **60728321** | **3.33** |  | *PRPF19* | Pre-MRNA Processing Factor 19 |
|  | 60751569 | 3.29 |  | *TMEM109* | Transmembrane Protein 109 |
|  | 60758280 | 3.29 |  | ***TMEM132A*** | **Transmembrane Protein 132A** |
|  |  |  |  | *SLC15A3* | Solute Carrier Family 15 Member 3 |
|  |  |  |  | *CD6* | CD6 Molecule |
|  |  |  |  | *CD5* | CD5 Molecule |
|  |  |  |  | *VPS37C* | VPS37C Subunit Of ESCRT-I |
|  |  |  |  | *PGA3* | Pepsinogen A3 |
|  |  |  |  | *PGA4* | Pepsinogen A4 |
| 11 |  |  | L-carnitine, Acylcarnitine C3:0 | *PTGDR2* | Prostaglandin D2 Receptor 2 |
|  |  |  |  | *ZP1* | Zona Pellucida Glycoprotein 1 |
|  |  |  |  | *PRPF19* | Pre-MRNA Processing Factor 19 |
|  |  |  |  | *TMEM109* | Transmembrane Protein 109 |
|  | 60801310 | 3.27 |  | ***TMEM132A*** | **Transmembrane Protein 132A** |
|  | 60825214 | 3.46 |  | *SLC15A3* | Solute Carrier Family 15 Member 3 |
|  | **60830260** | **3.75** |  | *CD6* | CD6 Molecule |
|  |  |  |  | *CD5* | CD5 Molecule |
|  |  |  |  | *VPS37C* | VPS37C Subunit Of ESCRT-I |
|  |  |  |  | *PGA3* | Pepsinogen A3 |
|  |  |  |  | *PGA4* | Pepsinogen A4 |
| 11 |  |  | L-carnitine | *DENND2B* | DENN Domain Containing 2B |
|  |  |  |  | *AKIP1* | A-Kinase Interacting Protein 1 |
|  | 9067368 | 3.56 |  | *C11orf16* | Chromosome 11 Open Reading Frame 16 |
|  | **9071106** | **3.58** |  | *NRIP3* | Nuclear Receptor Interacting Protein 3 |
|  | 9071236 | 3.44 |  | *SCUBE2* | Signal Peptide, CUB Domain And EGF Like Domain Containing 2 |
|  |  |  |  | *DENND5A* | DENN Domain Containing 5A |
|  |  |  |  | *TMEM14B* | Transmembrane Protein 14B |
| 14 |  |  | Acylcarnitines C10:0, 10:1 | *NOP9* | NOP9 Nucleolar Protein |
|  |  |  |  | *CIDEB* | Cell Death Inducing DFFA Like Effector B |
|  |  |  |  | *LTB4R2* | Leukotriene B4 Receptor 2 |
|  |  |  |  | *LTB4R* | Leukotriene B4 Receptor |
|  |  |  |  | *ADCY4* | Adenylate Cyclase 4 |
|  |  |  |  | *RIPK3* | Receptor Interacting Serine/Threonine Kinase 3 |
|  |  |  |  | *NFATC4* | Nuclear Factor Of Activated T Cells 4 |
|  |  |  |  | *NYNRIN* | NYN Domain And Retroviral Integrase Containing |
|  |  |  |  | *CBLN3* | Cerebellin 3 Precursor |
|  | 24836815 | 3.15 |  | *KHNYN* | KH And NYN Domain Containing |
|  | 24871290 | 3.17 |  | *SDR39U1* | Short Chain Dehydrogenase/Reductase Family 39U Member 1 |
|  | **24872997** | **3.65** |  | *IPO4* | Importin 4 |
|  |  |  |  | *NEDD8* | NEDD8 Ubiquitin Like Modifier |
|  |  |  |  | *GMPR2* | Guanosine Monophosphate Reductase 2 |
|  |  |  |  | *TINF2* | TERF1 Interacting Nuclear Factor 2 |
|  |  |  |  | *TGM1* | Transglutaminase 1 |
|  |  |  |  | *RABGGTA* | Rab Geranylgeranyltransferase Subunit Alpha |
|  |  |  |  | *DHRS1* | Dehydrogenase/Reductase 1 |
|  |  |  |  | *CMA1* | Chymase 1 |
|  |  |  |  | *CTSG* | Cathepsin G |
|  |  |  |  | *GZMH* | Granzyme H |
|  |  |  |  | *GZMB* | Granzyme B |
| 17 | **26125918** | **3.65** | Fatty acids C7:0 and 18:3 | *LGALS9* | Galectin 9 |
|  | 26127832 | 3.27 |  | *KSR1* | Kinase Suppressor Of Ras 1 |
|  | 26147411 | -3.10 |  | ***NOS2*** | **Nitric Oxide Synthase 2** |
|  |  |  |  | *LYRM9* | LYR Motif Containing 9 |
| 18 | 47017820 | 3.07 | L-carnitine | *RPL17* | Ribosomal Protein L17 |
|  | 47035065 | 3.32 |  | ***LIPG*** | **Lipase G, Endothelial Type** |
|  | **47086998** | **3.75** |  | *DYM* | Dymeclin |
| 19 |  |  | Fatty acid C18:3 | *ZNF507* | Zinc Finger Protein 507 |
|  |  |  |  | *DPY19L3* | Dpy-19 Like C-Mannosyltransferase 3 |
|  | 33004201 | 3.12 |  | *PDCD5* | Programmed Cell Death 5 |
|  | 33028578 | 3.09 |  | *ANKRD27* | Ankyrin Repeat Domain 27 |
|  | **33028651** | **3.23** |  | *RGS9BP* | Regulator Of G Protein Signaling 9 Binding Protein |
|  |  |  |  | ***NUDT19*** | **Nudix Hydrolase 19** |
|  |  |  |  | *TDRD12* | Tudor Domain Containing 12 |
