# Supplementary material for: Dysregulated Fatty Acid Metabolism in Preeclampsia Among Highland Andeans: Insights Into Adaptive and Maladaptive Placental Metabolic Phenotypes
Source: FASEB J. 2025 Nov 22;39(22):e71254. doi: 10.1096/fj.202502590R (PMC12639537; doi:10.1096/fj.202502590R)
Supplement: Supplementary file 6 — Appendix S1: fsb271254‐sup‐0006‐supinfo.docx. [file FSB2-39-e71254-s001.docx]

**Supplementary Figure 1: Abundance of L-carnitine or medium-chain fatty in normotensive (control) or preeclamptic (PE) pregnancy.** No difference observed for L-carnitine **(A)** or medium-chain fatty acids (FA) carbon chain lengths 6-12 **(B).** Peak intensities were normalized using pareto scaling and log transformation. Control and preeclamptic values were compared using an unpaired Student’s t – test. n = 14 control, 14 preeclamptic (PE, blue).

**Supplementary Figure 2: Comparison of fatty acid abundance in the umbilical cord venous plasma, separated by mono- or poly- unsaturated status. A)** Abundance of monounsaturated fatty acids (MUFAs), inclusive of C14:1, 16:1, 18:1; **B)** Abundance of polyunsaturated fatty acids (PUFAs), inclusive of C18:2, 18:3, 20:4, 20:5, 22:6, 20:3, 22:5; **C)** Ratio of MUFA/PUFA. Fatty acid abundance was determined from raw peak intensity values. Significant differences in metabolite abundance between control and preeclamptic pregnancies are indicated by an asterisk, *p<0.05. n = 14 control, 14 preeclamptic (blue).

**Supplemental Table 1:** Association analysis between acylcarnitines across all chain lengths in the cord plasma, placenta and maternal plasma and birthweight. Analysis performed using simple linear regression. Acylcarnitines (AC) are presented as carbon chain length: number of double bonds. Only significant values are presented. * significant with GA covariate. n = 14 control, 14 preeclamptic.

**Supplemental Table 2:** Association analysis between fatty acids across all chain lengths in the cord plasma and birthweight. Analysis performed using simple linear regression. Fatty acids (FA) are presented as carbon chain length: number of double bonds. Only significant values are presented. * significant with GA covariate. n = 14 control, 14 preeclamptic.

**Supplemental Table 3**: Fetal haplotypes associated with placental acylcarnitines, L-carnitine and fatty acids. Presented are genes within 200kb up and downstream of the top marker position. Highlighted in bold are the top iHS marker position and the prioritized gene within that region, based upon relevance to control of metabolic function the associated placental phenotype. Blue highlighted regions are presented in **Figure 3**.
